# VO_2_max (VO_2_peak) in elite athletes under high-intensity interval training: A meta-analysis

**DOI:** 10.1016/j.heliyon.2023.e16663

**Published:** 2023-06-01

**Authors:** Xianghua Ma, Zhenbo Cao, Zheng Zhu, Xiangru Chen, Donglin Wen, Ziwei Cao

**Affiliations:** a Shanghai University of Sport, China; b Xi'an Physical Education University, China

**Keywords:** Meta-Analysis, High-intensity interval training, Elite athlete, VO_2_max

## Abstract

Consensus is lacking regarding whether high-intensity interval training (HIIT) effectively improves VO_2_max (VO_2_peak) in elite athletes (Athlete must be involved in regular competition at the national level). This meta-analysis compared the effects of HIIT and conventional training methods (continuous training, repeated-sprint training, high volume low-intensity training, high-intensity continuous running, sprint-interval training, moderate-intensity continuous training)on VO_2_max in elite athletes. Nine studies were included, comprising 176 elite athletes (80 female). Compared to that with conventional training, VO_2_max was significantly increased after HIIT (overall: 0.58 [0.30, 0.87], I^2^ = 0.49, P = 0.03; males: 0.41 [0.06, 0.76], I^2^ = 0%, P = 0.89). VO_2_max had positive training effects when the HIIT recovery period had an interval time ≥2 min (0.44 [0.03, 0.84], I^2^ = 0%, P = 0.99) and recovery phase intensity ≤40% (0.38 [0.05, 0.71], I^2^ = 0%, P = 0.96). Thus, HIIT shows superiority over conventional training methods in improving VO_2_max, promoting aerobic capacity, in elite athletes.

## 1 Introduction

The ultimate goal of athletes and coaches is to obtain excellent performance. In order to stand on the highest podium, athletes must reach or create their highest level of sports performance. For elite athletes, knowing how to select and combine training programs to improve their sports performance is crucial.

Elite-athletes maintain a high level of training for a long time, at this point, routine training has limited effect on the improvement of capacity, so it is necessary to consider new training methods. Generally, the higher VO_2_max (VO_2_peak), the great aerobic and health. Did HIIT has a positive effect on VO_2_max (VO_2_peak) in elite athletes? If HIIT could enhance VO_2_max, how different HIIT composition methods (such as intensity, the length of intermittent recovery period, and the intensity of intermittent) produced different training effects. Coaches and athletes needed to “convince” of the importance of HIIT for VO_2_max. By study the effect of various possible factors of HIIT on VO_2_max of elite athletes, in order to provide help for high-level training.

In contrast to studies on elite athletes, most studies were conducted on non-elite athletes (adolescent athlete collegiate athlete), healthy adults, patients [[1], [2], [3], [4], [5], [6], [7]]. The results showed that HIIT were more easily to traditional training methods in improving VO_2_max, and has nothing to do with HIIT approach.

HIIT has improved anaerobic and aerobic capacity (VO_2_max), physical fitness in highly trained athletes [[8], [9], [10], [11]]. Ravier found that HIIT can significantly improve both the VO_2_max (4.6%) and anaerobic capacity (10.3%) of elite male Karate athletes [12]. During some rowing race, both aerobic and anaerobic energy systems are high demands [13]. For a long time, it was widely believed that aerobic exercise is an effective training method for improving aerobic capacity, while anaerobic exercise is an effective training method for improving anaerobic capacity [14]. However, when humans exercise for 2 min to exhaustion, oxygen deficiency reaches it maximum, which suggests that when the aerobic oxidation energy supply system is fully mobilized, the anaerobic energy supply system is also mobilized to its maximum. When humans exercise for 30 s to exhaustion, about 30% of the energy supply depends on the aerobic energy system; even after 10 s of exhausting exercise, 10% of the energy still comes from the aerobic system [15]. Therefore, when athletes meet a bottleneck in training, they can break through themselves through HIIT to improve their athletic ability.

Accordingly, there is no simple distinction between aerobic and anaerobic exercise [15], and Laursen thought that HIT can be defined as a repeated set of exercises of short to moderate duration completed at an intensity above the anaerobic threshold. In fact, exceeding the anaerobic threshold is an important basis for HIIT [16], which provides the theoretical basis for the popularity of HIIT worldwide. Compared to other training methods, HIIT is characterized by a short duration, time efficiency, high intensity, and small amount of exercise. However, the training effect can reach, or even exceed, that with conventional methods, and its unique training mode meets the training purposes of aerobic and anaerobic oxidation.

Numerous studies have shown that HIIT significantly improves athletic performance, and aerobic and anaerobic capacity [17]. Furthermore, HIIT effectively improves anaerobic capacity [[18], [19], [20]] and maximum oxygen consumption in athletes [18,21,22], and the lower limb training model of HIIT improves anaerobic and aerobic capacity [23,24]. Engel et al. found that HIIT not only improves the aerobic capacity of young athletes, but also promotes anaerobic capacity and sports performance [25]. Laursen and Jenkins reported that HIIT improves VO_2_max by approximately 6%–8% in high-level cyclists [26]. Gunnarsson found that HIIT improves the work efficiency by 3%–6% in elite soccer players [27]. Kinnunen et al. found that HIIT effectively improves neuromuscular efficiency and explosive power in ice hockey players [28]. Other studies found that HIIT improved athletes repeated sprint ability [21], sprint ability [22], ice speed [20], and vertical jump height [29]. Additionally, Monks et al. found that HIIT increased anaerobic capacity by 10.3% and aerobic capacity (VO_2_max) by 4.6% in elite karate athletes [2]. Christensen et al. found that the perfect design of HIIT can effectively improve the performance of elite athletes by 2%–3% [9].

Besides HIIT could promote adaptive changes in physiological indicators. Gibala et al. found that HIIT effectively promotes muscle and cardiovascular adaptive changes in athletes [30]. Clemente-Suárez et al. found HIIT training improves the functioning of the autonomic and parasympathetic nervous systems [31]. Some researchers found that HIIT was to be effective in improving VO_2_max [[32], [33], [34]] and running economy in endurance runners. This may be an increased oxidative capacity and a reduced plasma K^+^ concentration, which contributes to delay fatigue [[33], [34], [35]]. Compared with continue running, intensive running requires the activation of larger motor units, with increased recruitment of fast oxidative and glycolytic muscle-fiber, which contributes the contractile ability of the muscle [36,37]. Thus, regardless of the type of exercise, both aerobic and anaerobic energy supply systems are involved.

In addition to HIIT has been applied in various sports as an effective training method; specifically, some coaches have used HIIT to improve aerobic capacity [26,27,38]. However, some studies have suggested that HIIT impairs athletic performance. For example, Kingsley et al. suggested that HIIT could cause muscle damage and pain, as well as increase biomarkers of cell damage and lipid peroxidation [39]. Bloomer and Goldfarb found that HIIT induces lipid peroxidation, protein oxidation, and inflammatory responses, causing muscle cell damage and affecting the function of structural and contractile proteins [40]. These muscular disorders can affect the athlete's physiological function and athletic performance [41].

Although there are many studies on HIIT, most of them have focused on adolescents, sedentary populations, and patients. The results showed that HIIT improved the VO_2_max of these subjects more easily than traditional training methods [[1], [2], [3], [4], [5], [6], [7]]. Studies on HIIT in elite athletes are limited, and there is some controversy about the training effects of HIIT [[39], [40], [41]]. In addition, HIIT meta-studies on elite, high-level athletes are lacking. Therefore, we conducted a meta-analysis of the HIIT effect on high-level elite athletes in an effort to better understand the aerobic performance of elite athletes after HIIT; explore the possible training and physiological mechanisms of HIIT, to provide theoretical support for HIIT methods, with scientific insights for future studies.

## 2 Materials and methods

### 2.1 Search strategy

The systematic review was conducted applying the guidelines of PRISMA [42]. The following databases were searched from the time of their inception to March 2022: LISTA(37), PubMed(512), Web of Science(664), and China National Knowledge Internet(172). The following terms were used in the database searches: “high intensity interval training, high intensity training, intensive interval training, intensity training, sprint interval training, HIIT, HIT, IIT, or SIT” and “athlete”.

Intensity≥ 85%(PPO, HRmax, Pmax, VO_2_peak, MAP, vVO_2_peak).

HR, heart rate; HRmax, maximal HR; MAP, maximal aerobic power; PPO, power-output; Pmax, minimal power output to elicit VO_2_peak; vVO_2_peak, velocity of the VO_2_peak.

### 2.2 Inclusion criteria

Studies inclusion criteria were as follows: the study was a published experimental study; the study had a control training group; the study focused on high-level athletes; the HIIT training period was ≥3 weeks and the number of training session per week was ≥2 times; and the study specified the HIIT training intensity.

Elite athlete must be involved in regular competition at the national level.

### 2.3 Exclusion criteria

The study exclusion criteria were as follows: the study was a duplicate publication; the study population comprised adolescents and children; the study focused on drug interactions; the study focused on the combined effects of nutrition and tonics; the research object comprised patients; the study was a Masters or Doctoral dissertation; and the language is not English or Chinese.

### 2.4 Data extraction and risk of bias

In this paper, the data used are mean value and standard deviation from published journals. In one case, the data was obtained by contacting the corresponding author [43].

The PEDro scale was used to evaluate study quality and risk of bias. Two researchers evaluated the quality and risk of bias of the included studies in accordance with Sherrington C et al. [44]. The deviation risk for 11 items in the PEDro scale was evaluated as “yes”, “unknown”, or “no” for each included study. Differences of opinion were resolved by discussion with a third researcher.

The assessment criteria included: 1. Eligibility criteria were specified; 2. Subjects were randomly allocated to groups (in a crossover study, subjects were randomly allocated an order in which treatments were received); 3. allocation was concealed; 4. The groups were similar at baseline regarding the most important prognostic indicators; 5. There was blinding of all subjects; 6. There was blinding of all therapists who administered the therapy; 7. There was blinding of all assessors who measured at least one key outcome; 8. Measures of at least one key outcome were obtained from more than 85% of the subjects initially allocated to groups; 9. All subjects for whom outcome measures were available received the treatment or control condition as allocated or, where this was not the case, data for at least one key outcome was analyzed by “intention to treat”; 10. The results of between-group statistical comparisons are reported for at least one key outcome; 11. The study provides both point measures and measures of variability for at least one key outcome.

Studies were evaluated by classifying each study criterion as low risk of bias, moderate risk of bias, or high risk of bias. When Score ≦4, “high”, 4＜Score≦6, “moderate”, score≥7, “low”.

### 2.5 Statistical Analysis

The meta-analysis was performed using Review Manager 5.3 software. Homogeneity testing was conducted for included studies. The homogeneity was tested by chi-square test. When I^2^＜50%, and P > 0.05, homogeneity was considered. If the included studies were homogenous, the fixed-effect model was adopted; if there was heterogeneity, the random-effect model was adopted. Subgroup analyses were conducted to exclude heterogeneity. Combined statistical analysis was performed using the weighted mean difference (WMD) and 95% confidence interval (CI). The level of statistical significance was set at p < 0.05.

## 3 Results

### 3.1 Literature retrieval and selection

**Fig. 1:**
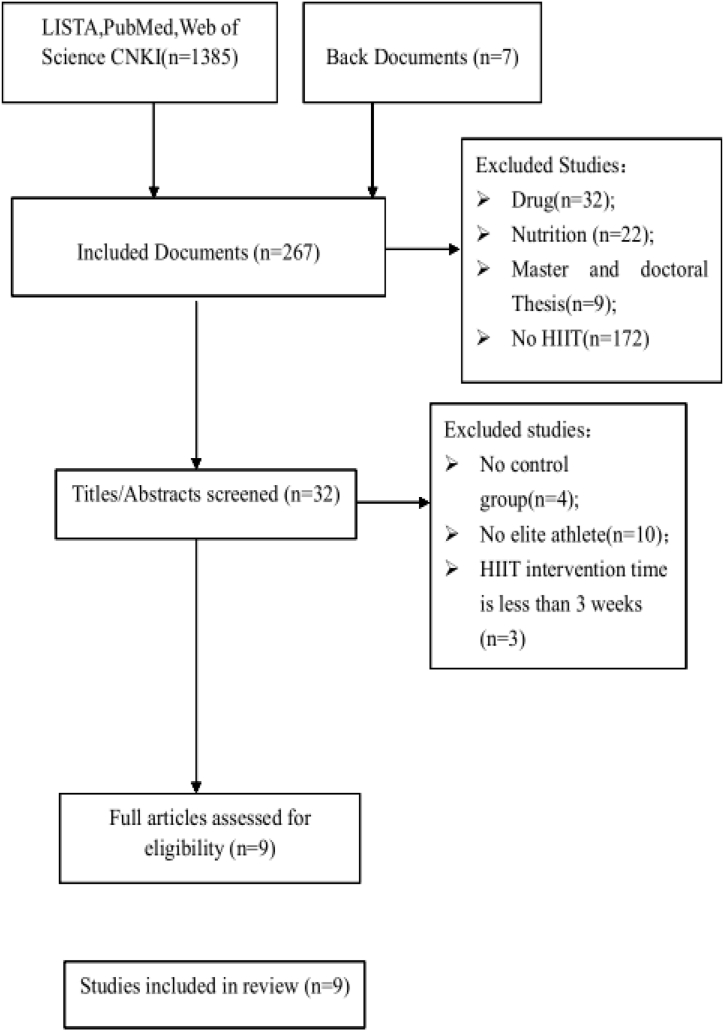
Study selection process.

In total, 1385 articles were identified in the database searches, among these, 51 of the papers were duplicates, 1067 studies were considered irrelevant because they included drugs (32), nutrition (22), master and doctoral dissertation(9), no HIIT(172). 1067 studies were considered irrelevant because of the combination of drug(32), nutrition(22), master and doctoral thesis and no HIIT. The titles and abstracts of the remaining 276 studies were screened, and the text of 32 studies was fully evaluated. Among these, 23 studies were excluded for following reasons: no control group (n = 4), not focused on elite athletes (n = 16), and HIIT intervention time <3 weeks (n = 3). Finally, a total of 9 studies met inclusion criteria, including 8 English studies and 1 Chinese study (Fig. 1).

The included studies comprised 176 high-level athletes in different sports (kayaking, tennis, taekwondo, triathlons, and cycling), including 80 female athletes. Athletes in the experimental group received HIIT, while those in the control group received traditional training for improving aerobic capacity. A detailed description of the training intensities is provided in Table 1.

**Table 1:** Basic characteristics of included studies.

Study	Characteristics				Training Information			Main Outcomes
	Sex M/F	Age (years)	Sport	Athlete level	Intensity	Weeks/Sessions	HIIT setting and protocol	
Ní- PAGE 24 - Chéilleachair et al., 2016	14/5	22 ± 4	Rower	National	HIIT: 100% PPO; Interval: 40% PPO CON: Aerobic;	8/16	HIIT: 1～2 weeks (6 intervals, 2.5 min in each session); 3～4weeks (7 intervals); 5～8weeks (8 intervals). Recovery: 40% PPO Rowing,	VO2max Greater improvement (ES = 0.95; P = 0.035)
Fernandez- Fernandez et al.,2012	11/0 9/0	HIIT: 22.6 ± 4.8; CON: 22.1 ± 3.3	Tennis	International tennis level-3	HIIT: 90～95% HRmax; Recovery: 70% HRmax CON: 22-m shuttle sprints;	6/18	HIIT: Running(3 sets, 90%–95% HRmax, 710-sec work, 540-sec rest); Recovery: Running (70% HRmax, 180-sec), Tennis game (8 min), 2:1 game.	VO2peak (+6.0%)
Laursen et al., 2002	8/0 9/0 10/0 11/0	HIITa: 26 ± 6 HIITb: 24 ± 7 HIITc: 25 ± 6 CON: 25 ± 5	Cycling triathlon	Highly trained	HIITa:Pmax; HIITb:Pmax; HIITc: 175% PPO; CON: Low to moderate intensity;	4/8	HIITa: Work (Tmax); rest (120% Tmax); 8 intervals (Pmax); HIITb: Work (Tmax); rest (65% HRmax); HIITc: Work (30 s); rest (4.5 min)	HIITa ：VO2peak(+5.4%) HIITb ：VO2peak(+8.1%)
Monks et al. 2017	8/8 9/8	HIIT: 18-22; CON: 18–22	Taekwondo	Elite	HIIT: 85%–100% HRmax; CON: 85% HRmax;	4/12	HIIT: Running (85%–100% HRmax), Recovery: Walking (60 s, rested for 120 s). CON: Continuous running for 5 km (85% HRmax).	HIIT：Greater improvement in aerobic capacity
Yang et al., 2017	7/0 7/0	HIIT: 18.1 ± 2.4; CON: 19.3 ± 2.1	Canoeing	National	HIIT: 90%VO2peak; CON: 65% VO2peak	4/12	HIIT: 90% VO2peak (100.7 ± 14.3 W, 84.0–124.0 W), 2 min, 7 bouts, 1 min rest. CON: 65%VO2peak (65.0 ± 13.1 W, 46.0–81.0 W), 20 min.	Power at VO2peak significantly improved
Qu et al.[73] 2019	5/0 5/0	HIIT: 21.8 ± 3.4; CON: 22.6 ± 4.3	Cycling	National	HIIT: 90%–100% MAP; Interval: 60%–70% HRmax; CON: 65%–75% HRmax	3/6	HIIT: 90%–100% MAP(4 min), Interval: 65%–75% HRmax(4 min)	Higher VO2max
Papandreou et al.[72].,2018	18/6	18.2 ± 4.1	Kayaking	National	HIIT: 120% VO2max; CON: Walking: 130 b·min-1	8/24	HIIT: 30-sec paddling(120% VO2max), Intervals: 60-sec walking (8 repetitions)	VO2max no change
Sheykhlouvand et al.[74] .2015	21/0	24 ± 3	Canoe polo	Professional	HIITa: 100% vVO2peak; HIITb: 100, 110, 120, 130, 130, 130, 120, 110, 100% vVO2 peak CON: 75% vVO2peak	3/9	HIITa: Paddling(1-min,6 times), Recovery(3 min, work to recovery, 1:3); HIITb: Paddling (1-min, 6 times) .	HIITa: VO2peak (+8.8%); HIITb: VO2peak (+8.5%)
Paquette et al. ,2021	HIIT: 4/2; CON: 4/2	21 ± 3 22 ± 4	Kayaking	National(5), Provincial(4), Club(3)	HIIT: 110% MAP, recovery:50% MAP; CON: 150 m to 200 m (30sec–40sec)	4/9	HIIT: 110% MAP (15-sec or 30-sec), Recovery: 50% MAP,1:1 effort:rest ratio.	$\dot{V}$O2peak no change

CON, control; ES, effect size; HR, heart rate; HRmax, maximal HR; HIIT, high-intensity interval training; MAP, maximal aerobic power; PPO, power output; Pmax, minimal power output to elicit VO2peak; Tmax, time to exhaustion at 60% Pmax; vVO2peak, velocity of the VO2peak.

**Fig. 2:**
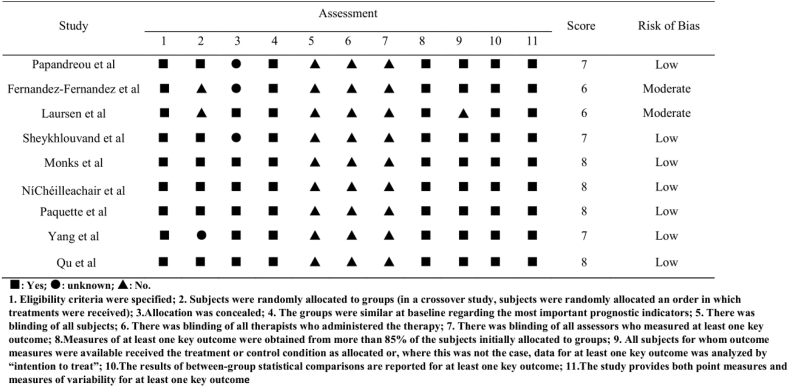
PEDro scale evaluation. Each of the selected studies was evaluated on each of the 11 items on the PEDro scale; “yes”, “unknown”, “no”, are indicated as “■“, “●“, “▲“respectively.

The average PEDro score of the included studies was 7.2/11, with scores ranging 6–8, indicating balanced literature scores. There were 6 low-risk cases and 3 medium-risk cases (Fig. 2).

### 3.2 Risk of bias

The final assessment of the risk of bias in the included studies is shown in Figuere3. All evaluated papers included a statement on the randomization method. There are 7 studies scored ≥“7”, as low risk of bias, and 2 studies scored “6”, as moderate risk of bias. With blinding of all subjects, with blinding of all therapists who administered the therapy, and with blinding of all assessors who measured at least one key outcome risk of bias predominated.

### 3.3 Effect of HIIT on VO_2_max in elite athletes

**Fig. 3:**
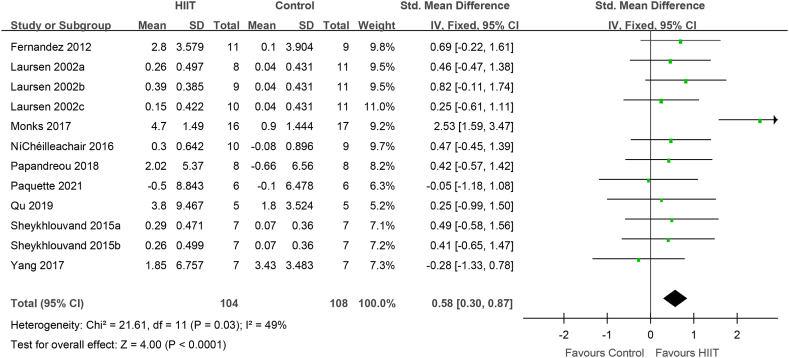
VO_2_max (VO_2_peak) in elite athletes: HIIT vs. conventional training (control). CI, confidence interval; HIIT, high-intensity interval training; SD, standard deviation.

VO_2_max (VO_2_peak) after HIIT was evaluated in 176 elite athletes from 9 studies (12 groups). Compared to that with conventional training, VO_2_max (VO_2_peak) was significantly increased after HIIT (0.58 [0.30, 0.87], P < 0.0001) (Fig. 3). Heterogeneity was observed in the included studies (I^2^ = 0.49, P = 0.03).

### 3.4 Subgroup analysis of the effect of HIIT on VO_2_max in male elite athletes

**Fig. 4:**
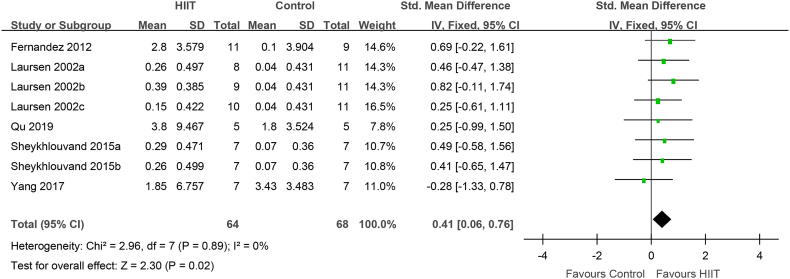
VO_2_max (VO_2_peak) in male elite athletes: HIIT vs. conventional training (control). CI, confidence interval; HIIT, high-intensity interval training; SD, standard deviation.

VO_2_max (VO_2_peak) after HIIT was evaluated in 96 elite male athletes from 5 studies. Compared to that with conventional training, VO_2_max(VO_2_peak) was significantly increased after HIIT in male athletes (0.41 [0.06, 0.76], P = 0.02) (Fig. 4). There was no heterogeneity in the included studies (I^2^ = 0%, P = 0.89).

### 3.5 Subgroup analysis of the effect of HIIT with recovery interval time ≥2 min on VO_2_max

**Fig. 5:**
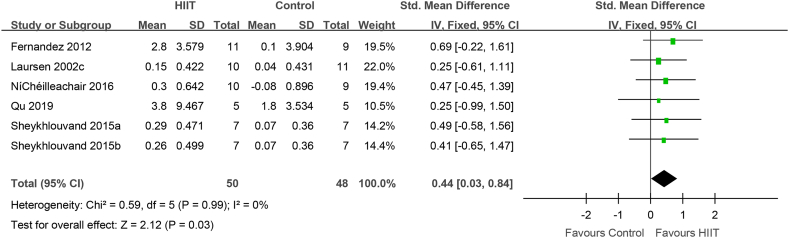
HIIT vs. conventional training (control) in studies with HIIT recovery interval duration ≥2 min. CI, confidence interval; HIIT, high-intensity interval training; SD, standard deviation.

Setting the HIIT recovery interval time as the research object, the effect of HIIT with recovery interval time ≥2 min was evaluated in 91 elite athletes from 5 studies. Compared to that with conventional training, VO_2_max (VO_2_peak) was significantly increased after HIIT with recovery interval ≥2 min (0.44 [0.03, 0.84], P = 0.03) (Fig. 5). There was no heterogeneity (I^2^ = 0%, P = 0.99).

Subgroup analysis of the effect of HIIT with recovery intermittent intensity ≤40%(PPO, HRmax, Pmax, VO2peak, MAP, vVO_2_peak) on VO_2_max

**Fig. 6:**
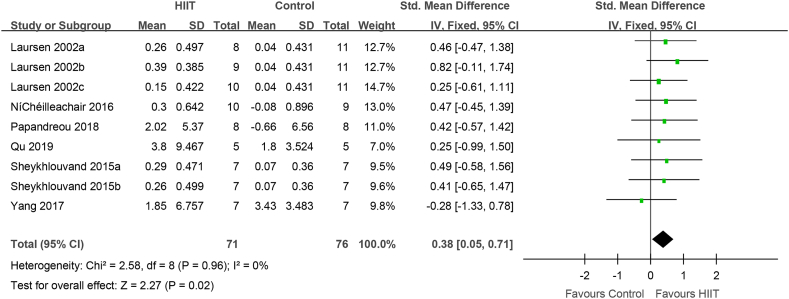
HIIT vs. conventional training (control) in studies with HIIT intermittent intensity ≤40%. CI, confidence interval; HIIT, high-intensity interval training; SD, standard deviation.

Setting the intermittent intensity of the HIIT recovery period as the research object, the effect of HIIT with recovery intermittent intensity ≤40% was evaluated in 111 elite athletes from 6 studies. Compared to that with conventional training, VO_2_max (VO_2_peak) was significantly increased after HIIT with recovery intermittent intensity ≤40% (0.38 [0.05, 0.71], P = 0.02) (Fig. 6). There was no heterogeneity (I^2^ = 0%, P = 0.96).

### 3.6 Subgroup analysis of the effect of HIIT against conventional training with intensity ≤75%

**Fig. 7:**
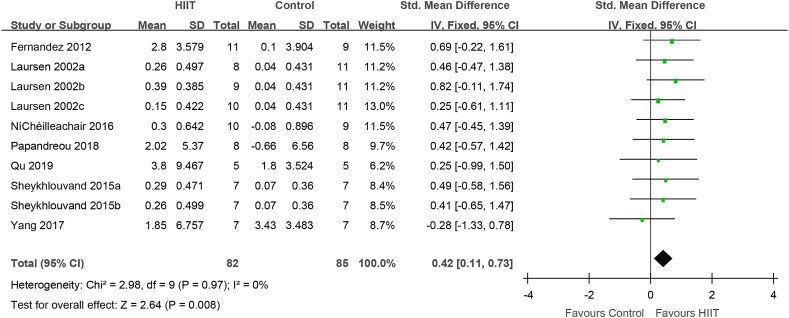
HIIT vs. conventional training (control) in studies with convention training intensity ≤75%. CI, confidence interval; HIIT, high-intensity interval training; SD, standard deviation.

Setting the control group intensity as the research object, the effect of HIIT against conventional training with intensity ≤75% was evaluated in 131 elite athletes from 7 studies. Compared to that with conventional training with intensity ≤75%, VO_2_max (VO_2_peak) was significantly increased after HIIT (0.42[0.11, 0.73], P = 0.008) (Fig. 7). There was no heterogeneity (I^2^ = 0%, P = 0.97).

### 3.7 Subgroup analysis of the effect of HIIT on taekwondo, tennis and kayaking athletes

**Fig. 8:**
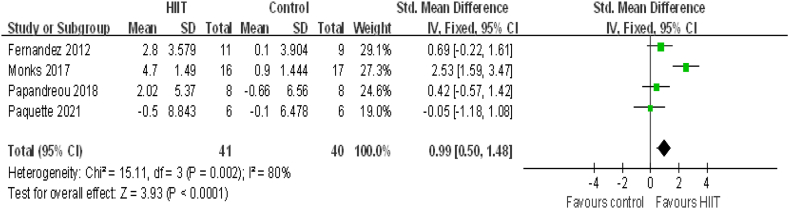
HIIT vs. conventional training (control) in studies on taekwondo, tennis and kayaking athletes. CI, confidence interval; HIIT, high-intensity interval training; SD, standard deviation.

Setting the taekwondo, tennis and kayaking athletes as the research object, the effect of HIIT on these was evaluated in 81 elite athletes from 4 studies. Compared to that with conventional training, VO_2_max (VO_2_peak) was significantly increased after HIIT (0.99[0.50, 1.48], P＜0.0001) (Fig. 8). There was no heterogeneity (I^2^ = 80%, P = 0.002).

### 3.8 Subgroup analysis of the effect of HIIT on other athletes. (No taekwondo, tennis and kayaking)

**Fig. 9:**
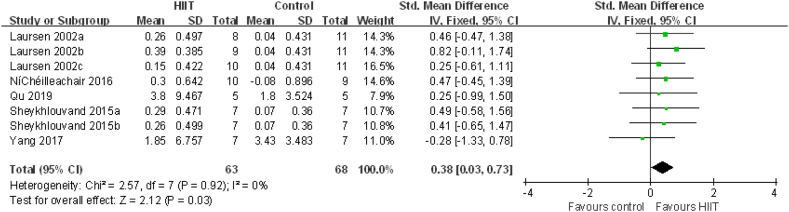
HIIT vs. conventional training (control) in studies on other athletes (no taekwondo, tennis and kayaking). CI, confidence interval; HIIT, high-intensity interval training; SD, standard deviation.

Setting other athletes (no taekwondo, tennis and kayaking) as the research object, the effect of HIIT on these was evaluated in 102 elite athletes from 5 studies. Compared to that with conventional training, VO_2_max (VO_2_peak) was significantly increased after HIIT (0.38[0.03, 0.73], P = 0.03) (Fig. 9). There was no heterogeneity (I^2^ = 0%, P = 0.92).

## 4 Discussion

### 4.1 HIIT and VO_2_max (VO_2_peak)

VO_2_max reflects the overall working status of cardiopulmonary and muscle oxidation functions [45,46], and is an effective index for evaluating endurance sports. In elite athletes, VO2max has reached a high level because of long-term planned training, and VO2max is greatly affected by genetic factors, so increases in VO2max are very small, however it is urgent for athletes to improve their ability, therefore, it is necessary to carefully select an effective training program to improve the physiological status of VO2max in elite athletes.

In the present meta-analysis, HIIT had a positive effect on VO_2_max in elite athletes (Fig. 3). Through the subgroup analysis of gender, intensity, the interval time and exercise program, it was found that the athletes were male, the recovery interval time was ≥2 min, the recovery interval intensity was ≤40%, the routine training intensity was ≤75%, except for taekwondo, tennis and kayaking athlete, VO2max was enhanced (Figs. 4–7,9). When the object of study were taekwondo, tennis and kayaking athletes, Although HIIT was found to have an effect on VO2max in such athletes, it was not statistically significant because the analysis results were heterogeneous, but according to the original literature, HIIT has been shown to increase VO2max such athletes. The reason for the heterogeneity may be that after HIIT training, the VO2max of taekwondo athletes (4.7 ± 1.49 ml/kg/min) increased more than that of tennis(2.8 ± 3.579 ml/kg/min) and kayaking athletes(2.02 ± 5.37 ml/kg/min). From the results, some HIIT scheme could facilitate VO_2_max.Therefor, if athletes can choose the most appropriate HIIT that can promote higher VO_2_max

How did HIIT contribute to the increase VO_2_max? IT is possible that HIIT increased cardiac output, thereby boosting VO_2_max. Fernandez-Fernandez et al. found that achieving and maintaining a high filling pressure in the heart is necessary to improve cardiac function [47]; thus, training at the maximum intensity cardiac output per stroke is important. Studies have found that before fatigue, the cardiac output per stroke reaches its peak during exercise in approximately 1 min [48], 2 min [18,49], or 4 min [50]. Thus, to improve the stroke volume in athletes, the duration of each HIIT interval should not be < 60sec. The evidence for short-duration (<60sec) HIIT in improving cardiac stroke volume is limited，however, short-duration HIIT may limit the effectiveness of the venous muscle pump, which in turn limits the maintenance of each stoke volume during the recovery period [51]. Furthermore, Nybo et al. suggested that the difference between the cardiac output and arterial venous oxygen concentration (a-vDO_2_) may determine VO_2_max [52]. Macpherson et al. found that although VO_2_max increased by 12% after a 6-week sprint interval training program, the maximum cardiac output did not change, which might underlie improvements in peripheral a-vDO_2_ [53]. Above all, HIIT increases the recruitment of rapid oxidation and glycolytic muscle fibers, which contribute to muscle contraction capacity, and changes in muscle contractility promote venous blood return and maintain cardiac output. In addition, varying the type of exercise, intensity, and recovery time during intervals can also help boost cardiac output. Nybo and Warburton found that, in patients and the general population, the maximum stroke volume occurred during the intermittent recovery period of HIIT [52,54]. The results of Buchheit, Racinais, Girard, and Laursen in elite cyclists are consistent with those of Cumming and Takahashi [53]. Similarly, Iaia and Bangsbo found that, regardless of the type of exercise (incremental load test, 3-min pVO2max test, or 15-s sprint run test), the maximum amount of exercise per stroke occurred during the intermittent recovery period [55]. Therefore, the intermittent recovery mode of HIIT can enhance the adaptability of cardiovascular function to sports in athletes. Although VO2max may be determined by the effect of the intensity and form of HIIT intermittent recovery on the stroke volume, Stanley and Buchheit et al. found that the stroke output was the same for HIIT intermittent recovery periods of 0%, 30%, and 60% VO2max intensity in elite cyclists [30]. In addition, during HIIT interval recovery, a long interval (>2 min) is conducive to maximizing the stroke volume [30,56]. The present meta-analysis found that HIIT with interval recovery duration ≥2 min and intensity ≤40% improved the VO_2_max in elite athletes (Fig. 5, Fig. 6), which may promote improved cardiac output and peripheral aerobic metabolism capacity.

Illi et al. found that the training intensity promoted the oxidative adaptability of muscle fibers only when the HIIT intensity was close to VO_2_max [57]. Under this condition, the recruitment of motor units, maximum ventilation rate, and cardiac output jointly induced VO_2_max [58]. Laursen and Jenkins found that effective physiological stimulation only occurs when the HIIT training intensity is at least 90% VO_2_max and is sustained for several minutes, which could increase VO_2_max [59,60]. In the present meta-analysis, the HIIT intensities of the studies that showed improvement in VO_2_max included 100% all-out power, >90–95% maximal heart rate (HRmax), minimal power output to elicit VO_2_peak, 85%–100% HRmax, 90%–100% maximal aerobic power, 100% velocity of the VO_2_peak, and ≥90% VO_2_max, with training times that could be > 200 s.

Effective training methods can promote improvement in VO2max in athletes. According to specific training theories and the SAID principle, which asserts that the human body adapts specifically to applied demands, specific HIIT protocols can promote improvement in athletes' physiological function for different sports [30,61]. Based on this principle, training methods similar to one's own sport should be selected, and athletes should repeatedly-training within their own limits. To promote the adaptability of the physiological system and achieve training effects, HIIT is the perfect method for specific training needs and applications.

### 4.2 HIIT and aerobic and anaerobic capacity

HIIT may promote improvement in aerobic capacity via various mechanisms, such as increased ventilation threshold [62]; enhanced mitochondrial enzyme activity [50], lipid oxidation [63], and H+ elimination ability; increased glycogen utilization and reduced lactic acid accumulation [50]; and improved cardiovascular function (enlarged heart, increased blood flow capacity, and arterial dilation) [64,65]. These adaptive changes in human physiology improve the ability of the cardiovascular system to transport oxygen, thus promoting improvements in human aerobic capacity [64].

HIIT can also improve anaerobic capacity in elite athletes. Studies have found that HIIT improves the anaerobic ability of athletes by 12% [8,17,66]. HIIT may impose greater load intensity on muscles, resulting in the promotion of rapid contraction muscle development [29]; enhanced glycolysis capacity [62] and glycolysis enzyme activity (lactate dehydrogenase, phosphofructokinase, glycogen phosphorylase) [67]; accelerated energy conversion rate of glycolysis [47]; increased number of myo-membrane transporters [48]; and enhanced muscle buffering capacity [68].

From the above we can know that HIIT can not only improve the VO2max of endurance exercise, but also improve the VO2max of anaerobic capacity exercise.

This may be HIIT improves aerobic and anaerobic capacity in different ways. Improvement in aerobic capacity is more about the development of heart function and slow contraction muscle, while the regulation of anaerobic capacity is more about fast twitch contraction muscle. Thus, HIIT may promote improvement in the aerobic capacity of fast twitch muscle and the anaerobic capacity of slow twitch muscle. Suitable HIIT methods can regulate and improve the proportion of ATP and ADP in the body. The accumulation of metabolites and free radicals in muscle; glycogen reserve; and rates of carbohydrate oxidation, mitochondrial content, and fat oxidation, GLUT4 transporter, glycogen content, and oxidative fiber conversion are increased with HIIT [17]. The synthesis efficiency of mitochondrial protein is also improved. All of these features promote both aerobic and anaerobic capacity.

### 4.3 Possible mechanisms of different HIIT combination modes

Different HIIT protocols (differing in intensity, duration, number of sessions, number of intervals, and mode of rest) can affect specific metabolic pathways and neuromuscular functions in muscle cells. Buchheit and Laursen suggested that acute physiological changes after different HIIT methods could be described based on three metabolic energy supply modes (ATP/CP, glycolysis, and oxidative metabolism) and the load status of the nervous skeletal muscle system [69]. The oxidase pathway of aerobic metabolism and the glycolytic enzyme system (ATP) of anaerobic metabolism are significantly upregulated after different forms of HIIT [68,70,71]. Therefore, priority should be given to aerobic ability training, with anaerobic ability training as an auxiliary method, which is often the more preferred mode of training for coaches and athletes. The advantage of anaerobic ability training is to promote better resistance against fatigue. The priority with aerobic exercise, with nerve muscle stress as the auxiliary role of training, is to improve the overall physical ability. In practice, in order to achieve the best training effect, the coach must be clear about the training purpose and select the HIIT mode with a clear target.

### 4.4 Limitations

This review has several limitations. First, a total of 9 articles were included in this paper, among which 4 articles did not divide the research objects between genders. Moreover, as the research objects were high-level athletes, the sample size of the 7 articles was small (<10). Second, the included studies used different HIIT regimens (exercise style, interval time, intensity, duration). The purpose of this paper is to provide the best evidence in HIIT practice, and our results justify the need for more rigorous research with great methods and larger sample sizes to strengthen the quality of existing evidence and determine HIIT most positively affect VO2max in elite athletes.

## 5 Conclusions

Accumulating studies have evaluated HIIT methods in the training practices of athletes. Compared to that with other training methods, HIIT can slightly improve VO2max and sports performance, and promote improvement in aerobic and anaerobic capacity. Considering the training specificity of elite athletes, the value of HIIT training is worthwhile. During practice, coaches should reasonably select the HIIT form according to the training purpose.

### 5.1 Practical implications

This report comprises the first meta-analysis of the HIIT effect in elite athletes. VO2max (VO2peak) was increased after HIIT in elite athletes. For excellent endurance and to better enhance the training effect, athletes should undergo not only aerobic ability training, but also anaerobic ability training. The recovery intensity and duration of HIIT play important roles in the cardiopulmonary function of endurance athletes.

## Funding

This study was supported by the Science and Technology Department of Shaanxi Province, China (grant no. 2021JM-529).

## Production notes

### Author contribution statement

All authors listed have significantly contributed to the development and the writing of this article.

### Data availability statement

Data included in article/supplementary material/referenced in article.

### Additional information

No additional information is available for this paper.

## Declaration of competing interest

The authors declare that they have no known competing financial interests or personal relationships that could have appeared to influence the work reported in this paper.
